# Genetic and epigenetic characterization of the tumors in a patient with a tongue primary tumor, a recurrence and a pharyngoesophageal second primary tumor

**DOI:** 10.1186/s13039-017-0310-z

**Published:** 2017-04-11

**Authors:** Ilda P. Ribeiro, Francisco Marques, Leonor Barroso, Jorge Miguéis, Francisco Caramelo, André Santos, Maria J. Julião, Joana B. Melo, Isabel M. Carreira

**Affiliations:** 1 Cytogenetics and Genomics Laboratory, Faculty of Medicine, University of Coimbra, Polo Ciências da Saúde, Coimbra, 3000-354 Portugal; 2 CIMAGO - Center of Investigation on Environment Genetics and Oncobiology - Faculty of Medicine, University of Coimbra, Coimbra, 3000-354 Portugal; 3 Department of Dentistry, Faculty of Medicine, University of Coimbra, Coimbra, 3000-075 Portugal; 4 Stomatology Unit, Coimbra Hospital and University Centre, CHUC, Coimbra, 3000-075 EPE Portugal; 5 Maxillofacial Surgery Department, Coimbra Hospital and University Centre, CHUC, Coimbra, 3000-075 EPE Portugal; 6 Department of Otorhinolaryngology - Head and Neck Surgery, Coimbra Hospital and University Centre, CHUC, Coimbra, EPE Portugal; 7 Laboratory of Biostatistics and Medical Informatics, IBILI - Faculty of Medicine, University of Coimbra, Coimbra, 3000-354 Portugal; 8 Department of Pathology, Coimbra Hospital and University Centre, CHUC, Coimbra, 3000-075 EPE Portugal

**Keywords:** Recurrence, Second primary tumor, Genetic and epigenetic profile, Oral cancer, Chemoradioresistance

## Abstract

**Background:**

The choice of therapeutic modality for oral carcinoma in recurrent or second primary tumors remains controversial, as the treatment modalities available might be reduced by the treatment of the first tumor, and the overall survival is lower when compared with patients with a single or first tumor. Identifying biomarkers that predict the risk of relapse and the response to treatment is an emerging clinical issue.

**Case presentation:**

A Caucasian 49-years-old man was treated with chemotherapy followed by chemoradiotherapy for a primary left side tongue tumor, achieving a complete response. After 49-months of follow-up, a local recurrence was diagnosed. After 3 months, a second primary tumor at the pharyngoesophageal region was detected. Genomic and epigenetic characterization of these three tumors was performed using array Comparative Genomic Hybridization, Multiplex Ligation-dependent Probe Amplification (MLPA) and Methylation Specific MLPA.

**Results:**

The three tumors of this patient shared several imbalances in all chromosomes excluding chromosomes 9, 20 and 22, where genes related to important functional mechanisms of tumorigenesis are mapped. The shared genomic imbalances, such as losses at 1p, 2p, 3p, 4q, 5q, 6q, 7q, 8p, 10p, 11q, 12p, 12q, 13q, 15q, 16p, 16q, 17p, 17q, 18q, 19p, 19q, 21q and Xp and gains at 3q, 7q, 14q and 15q showed a common clonal origin for the diagnosed relapses.

We identified some chromosomal imbalances and genes mapped in the chromosomes 2, 3, 4, 6, 7, 11, 14, 17, 18 and 22 as putative linked to chemoradioresistance and chemoradiosensitivity. We also observed that gains in short arm of chromosomes 6, 7, 8 and 18 were acquired after treatment of the primary tumor. We identified losses of *VHL* gene and promoter methylation of *WT1* and *GATA5* genes, as predictors of relapses.

**Conclusions:**

A common clonal origin for the diagnosed relapses was observed and we identified some putative candidate biomarkers of prognosis, relapse risk and treatment response that could guide the development of management strategies for these patients.

## Background

Oral squamous cell carcinoma (OSCC) is the most common malignant tumor of the head and neck [1]. These tumors are associated with high morbidity and mortality [2] and their incidence is increasing in the younger population [3]. Considering all tumors that arise in the head and neck region, tongue tumors are among the worst in terms of prognosis [4]. Treatment of OSCC is predominantly based on tumor location and TNM classification and includes surgery, radiotherapy and chemotherapy, either individually or in combination [5]. These treatment modalities do not benefit patients equally and are often associated with side effects that reduce compliance and prevent timely completion of therapy [6]. OSCC survivors have a high risk of developing relapses (tumor recurrences or second primary tumors (SPT)) and also distant metastasis, which leads to treatment failure and hampers the overall survival [7]. The 5-years survival rate and disease-free survival of OSCC patients are negatively affected by the presence of recurrences, which lead to a poor prognosis and a poor quality of life. Since local recurrence and treatment resistance are the major obstacles in achieving a cure in this neoplasm, the identification of molecular markers to predict the risk of relapse development and the response to the treatment is important in the management of these patients. We report a Caucasian 49-years-old man diagnosed with a primary squamous cell carcinoma in the left side of the tongue. 49-months after the completion of treatment a local recurrence was diagnosed followed in the next 3 months by a second primary tumor at the pharyngoesophageal region. Genomic and epigenetic studies were conducted allowing the identification of shared imbalances by these three tumor samples in several chromosomal regions and genes, which could indicate a common clonal origin.

## Case presentation

### Sample 1 - primary tumor

In January 2011, a Caucasian 49-years-old man, drinker and heavy smoker (≥20 cigarettes/day), was diagnosed at the Maxillofacial Surgery and Stomatology Unit, of the Coimbra Hospital and University Centre, CHUC, EPE, Portugal, with a primary squamous cell carcinoma in the left side of the tongue. The diagnosis was confirmed by a biopsy and the well differentiated tumor was classified in advanced stage (IVa), cT4, cN2, cM0. Microscopically, the hematoxylin-eosin staining demonstrated the tumor formed by polygonal-shaped cells with eosinophilic cytoplasm and mild nuclei showing pleomorphism and hyperchromatic chromatin (photomicrograph unavailable). The treatment was three-cycles of chemotherapy (cisplatin 75mg/m2 x 1day, docetaxel 75mg/m2 x 1 day and 5-fluorouracil 1000mg/m2 x 5 days, on days 1, 22 and 43)) followed by three-cycles of chemoradiotherapy (cisplatin 75mg/m2 before the radiation – 60Gy/30 fractions on days 1, 22 and 43). One month after the completion of treatment the patient did not have any sign of neoplasm, achieving an apparent clinical and radiological complete response as evaluated by computed tomography scan. In July 2015, 49-months after the conclusion of treatment, the clinician observed a small suspicious lesion (5mm) in the left side of the tongue, in the same localization of the primary tumor.

### Sample 2- recurrence of the primary tumor

**Fig. 1:**
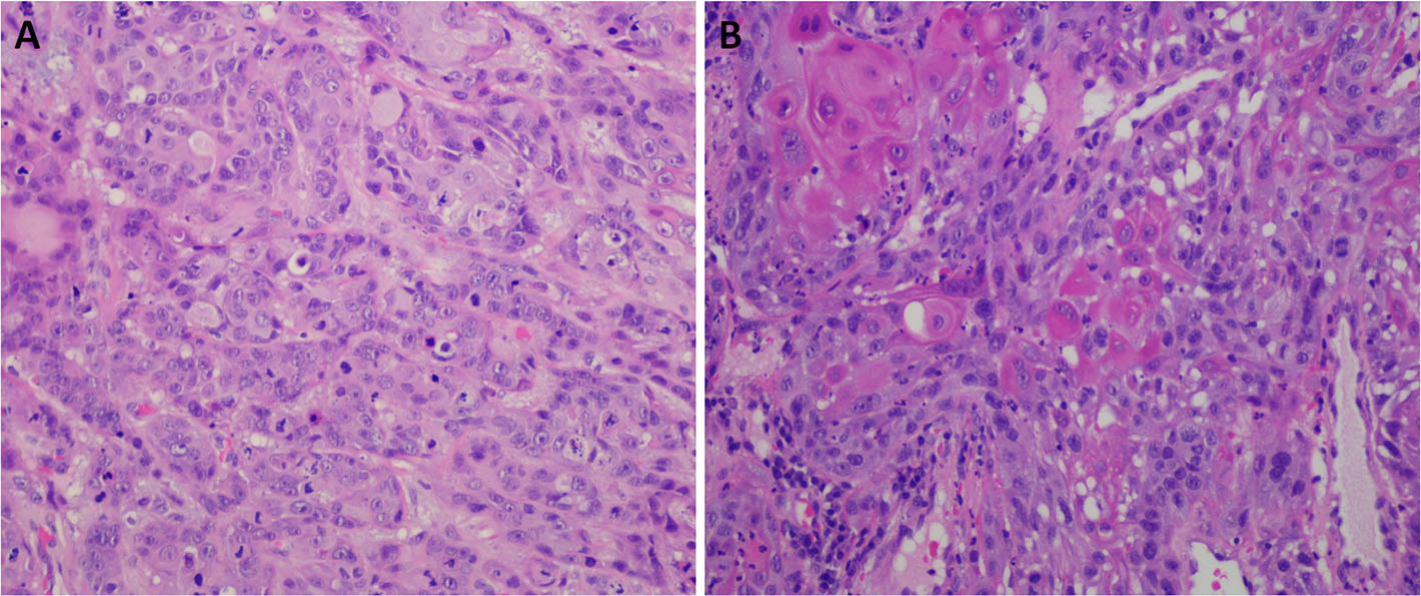
Hematoxylin-eosin stains showing the morphology of tumor cells (H&E 200x). **a** recurrence, **b** Second primary tumor (SPT)

In September 2015, a squamous cell carcinoma recurrence of the primary tumor in the left side of the tongue was diagnosed in this patient, according to the Warren and Gates criteria [8]. In October 2015, the patient underwent surgery and the tumor was completely removed. Microscopically, the hematoxylin-eosin staining showed tumor cells with eosinophilic cytoplasm, pleomorphic nuclei and numerous mitoses (Fig. 1a). The tumor showed a high mitotic index and areas of necrosis with vascular and neural invasion. All resection margins were negative for neoplastic involvement. The tumor was classified as early stage (II), rpT2, cN0, cM0. The patient stopped his smoking habits after the primary tumor diagnosis but kept the alcohol consumption.

### Sample 3- second primary tumor

In December 2015, a second primary tumor at the posterior wall of the pharyngoesophageal junction was diagnosed at the Department of Otorhinolaryngology - Head and Neck Surgery, Coimbra Hospital and University Centre, CHUC, EPE, Coimbra, Portugal. The diagnosis was confirmed by a biopsy and the well differentiated squamous cell carcinoma was classified in advanced stage (IVa), cT4b. Microscopically, the hematoxylin-eosin stain demonstrated a presence of dyskeratotic cells, polygonal-shaped cells with eosinophilic cytoplasm and nuclei showing mild to moderate atypia (Fig. 1b). The therapeutic decision was palliative care. In April 2016 the patient died.

### Genomic and epigenetic study

This study was approved by the Committee on Ethics in Research of the Faculty of Medicine of the University of Coimbra and written informed consent from the patient was obtained, performing all the experiments according to the regulations in the Declaration of Helsinki. Tumor tissue samples were obtained of the primary tumor and SPT from biopsies and of recurrence from the surgical resection. Additionally, macroscopically tumor-free tissue was also obtained from recurrence and SPT. This tissue, in the case of recurrence was collected from surgery resection margin and from an identical distance of the tumor in the case of SPT. The tissue samples were immediately snap-frozen in liquid nitrogen after resection and stored at −80 °C until use. DNA from fresh frozen tissues was extracted using a High Pure PCR Template Preparation Kit (Roche GmbH, Mannheim, Germany), according to the manufacturer’s instructions. We analyzed copy number alterations (CNAs) of the three tumor samples through array Comparative Genomic Hybridization (aCGH) using Agilent SurePrint G3 Human Genome microarray 180K, (Agilent technologies, Santa Clara, CA) as previously described [9]. Multiplex Ligation-dependent Probe Amplification (MLPA) and Methylation Specific MLPA (MS-MLPA) using the P248 and ME002 SALSA probemixes (MRC-Holland, Amsterdam, The Netherlands) were performed in tumor and non-tumor tissue samples in order to simultaneously evaluate the CNAs and methylation patterns in a specific set of genes as we previously described [10, 11]. DNA from gender-matched gingival tissue of healthy subjects submitted to wisdom teeth removal was used as controls. The same three controls were used for MS-MLPA and MLPA techniques and one of those controls was used for aCGH technique.

## Results and discussion

**Fig. 2:**
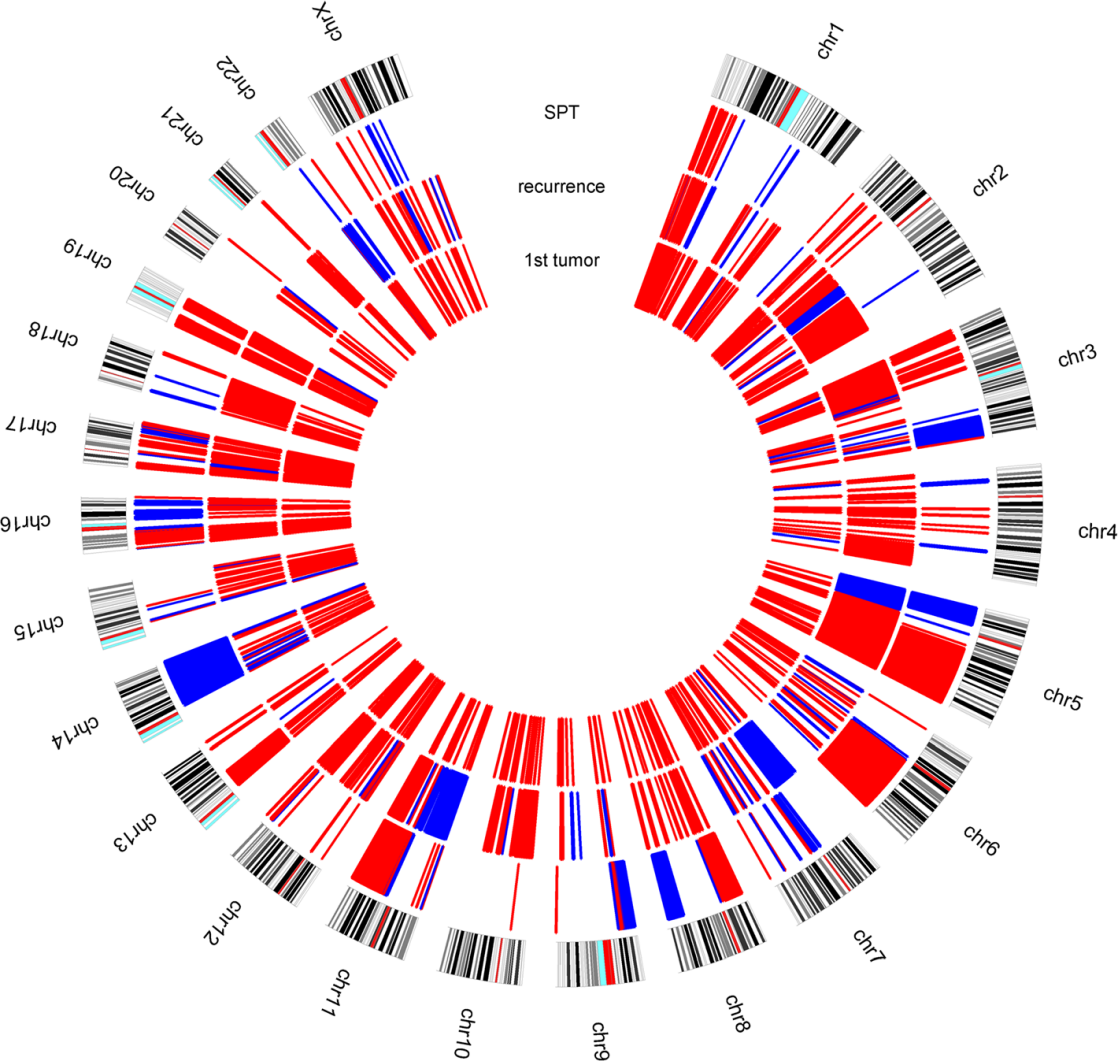
Circus plot revealing aberration pattern differences among the three tumor samples: primary tumor, recurrence and second primary tumor (SPT). Blue represents copy number gains and red copy number losses

**Fig. 3:**
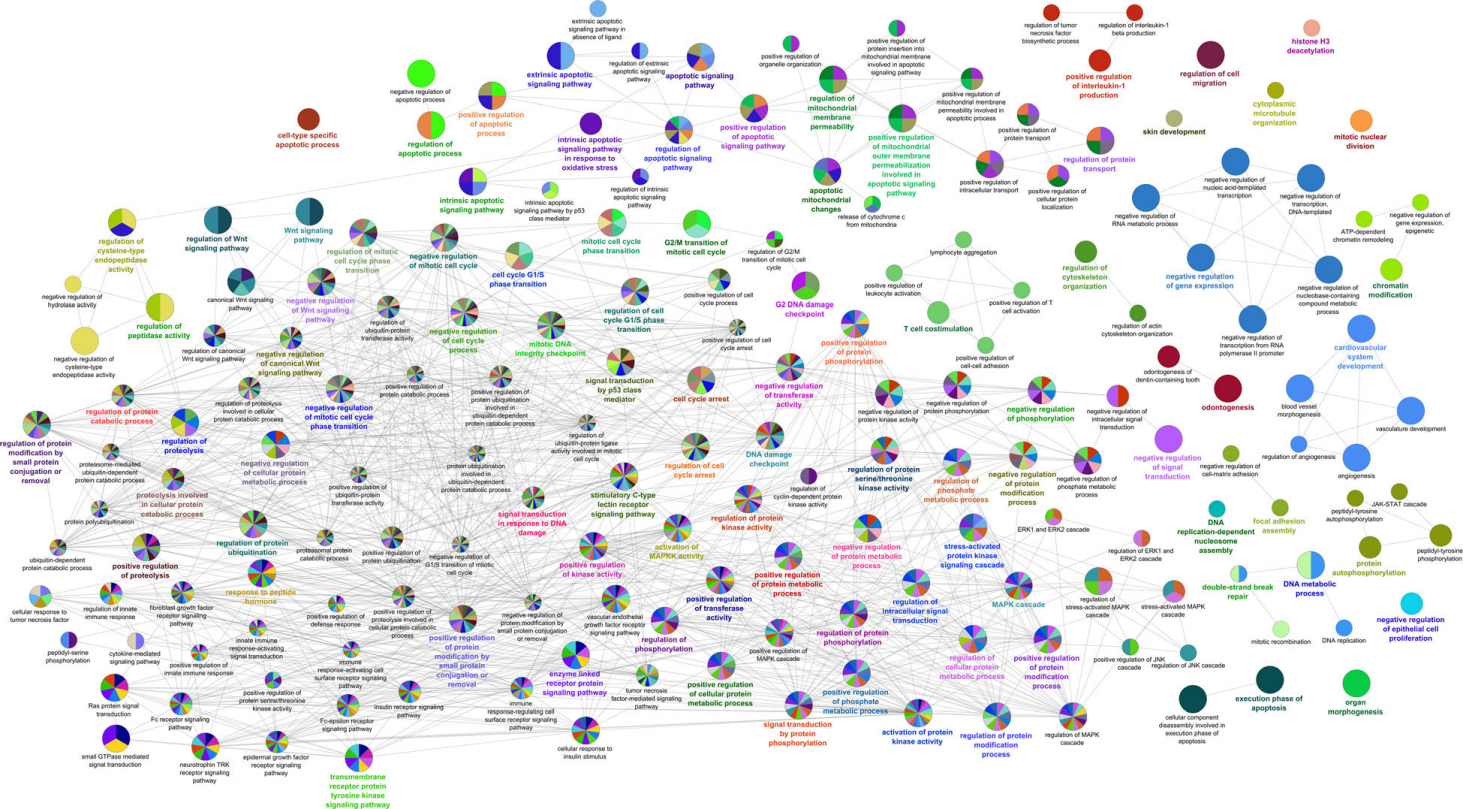
Genes simultaneously altered in the three tumor samples grouped in a functionally network that were linked to their biological function, highlighted regulation of apoptosis, cell cycle, cell proliferation, cell migration, angiogenesis, chromatin remodeling, DNA repair and ubiquitination (ClueGO analysis using Cytoscape). The ClueGO network is created with kappa statistics and reflects the relationships between the terms based on the similarity of their associated genes

When we compared the primary tongue tumor with recurrence and SPT, we found that these three tumors shared several genomic imbalances in almost all chromosomes, excluding chromosomes 9, 20 and 22 (Fig. 2). The simultaneous altered genes identified in these three tumor samples are linked with several cellular processes, namely regulation of apoptosis, cell cycle, cell proliferation, cell migration, angiogenesis, chromatin remodeling, DNA repair and ubiquitination (Fig. 3). These shared genomic imbalances seem to indicate that these three tumors have arisen from a common cell clone. Regarding the imbalances associated with chemoradioresistance and chemoradiosensitivity by Van den Broek and colleagues [12], we observed that in terms of size our samples presented some smaller chromosomal imbalances than those described (Table 1), which allow us to suggest some putative candidate genes in these specific chromosomal regions with an apparent link to radiotherapy response (Table 1). However, studies addressing the role of these genes in the chemoradioresistance and chemoradiosensitivity are needed. Our samples exhibited more imbalances associated with chemoradioresistance than chemoradiosensitivity. We could hypothesized that the recurrence evolved after the chemoradiotherapy treatment of the primary tumor, due to the presence of chemoradioresistance clone cells. Additionally, some imbalances of the recurrent and SPT cells seem to be further acquired after treatment, such as in short arm of chromosomes 6, 7, 8 and 18. Only the tumor from recurrence, diagnosed at stage II, presented simultaneous losses at 3p, 9p and 17p, the first imbalances described as associated with early tumor stage, by Califano and colleagues [13] (Table 2). All three tumor samples presented imbalances linked to poor patient outcome, being in the recurrence and SPT samples more evident the putative biomarkers of poor survival and of the presence of metastasis, namely gains in *EGFR* gene and at 11q13.3 [14–38] (Table 2).

**Table 1:** Chromosomal regions described in the study of Van den Broek et al. [12] as associated with chemoradioresistance and chemoradiosensitivity and the specific alterations and putative candidate genes identified in the present study related to those already described

			Present study			
Chromosomal region	Type of alteration	Clinical association	Primary tumor	Recurrence	SPT	Possible candidate genes
3q21-q26.1	Gain	Chemoradioresistance	3q26.1	3q26.1	3q26.1	*ZIC1, ZIC4*
6p11-pter				6p25.3-p25.2		*IRF4, HUS1B, FOXQ1, FOXC1, NQO2*
				6p21.33		*CYP21A2, TNXB, STK19*
				6p12.1		*GFRAL, HMGCLL1, BMP5*
					6p11.2	*PRIM2*
3q24	Amplification		3q24	3q24	3q24	-
7p11.2-12				7p12.1-p11.2	7p12.1-p11.2	*EGFR*
8p11.1					8p11.1	-
18p11.3					18p11.31-p11.23	*LAMA1, PTPRM*
3p11-pter	Loss		3p26.1	3p26.1	3p26.1	*ARL8B*
			3p25.3	3p25.3	3p25.3	BRPF1, CIDEC, FANCD2, IRAK2, SEC13, TADA3, VHL
			3p24.3	3p24.3		*EFHB, RAB5A, C3orf48*
			3p22.3	3p22.3		*TRIM71, CCR4*
			3p22.2	3p22.2	3p22.2	*MLH1*
			3p21.31	3p21.31	3p21.31	DHX30, CDC25A, ATRIP, TREX1, SHISA5, PFKFB4, NCKIPSD, IP6K2, ARIH2, KLHDC8B, APEH, UBA7, RBM5
			3p14.3	3p14.3	3p14.3	*APPL1, ARF4, ARHGEF3, DNASE1L3, FLNB, HESX1, IL17RD, LRTM1, WNT5A*
				3p13	3p13	*FOXP1, PPP4R2*
4p11-pter			4p16.3	4p16.3		*MXD4, TNIP2, NOP14*
			4p14	4p14		*UGDH, UBE2K*
11q distal			11q22.3	11q22.3	11q22.3	*ATM*
			11q23.3	11q23.3	11q23.3	TAGLN, KMT2A, CBL, H2AFX
17p13.1 (*TP53)*			17p13.1	17p13.1	17p13.1	*TP53*
14q distal	Gain	Chemoradiosensitivity	14q32.33	14q32.33	14q32.33	*-*
17q					17q24.1-q24.2	*PRKCA*
					17q25.1	*TTYH2*
22			22q11.23	22q11.23	22q11.23	*GSTT1*
2q31	Amplification		2q31.1		2q31.1	*-*
7q21				7q21.3	7q21.3	*PEG10*
14q13				14q13.3	14q13.3	*PAX9*
2q22-q25	Loss		2q24.2	2q24.2		*MARCH7, CD302*
7q11-q22			7q11.22 - q11.23	7q11.22 - q11.23		*WBSCR22, CLDN3*

**Table 2:** Several genomic imbalances identified in the three tumor samples and their clinical association described in the literature

					Present Study		
Chr.	*Genes*	Type of alteration	Clinical association	References	Primary Tumor	Recurrence	SPT
3p21.31	*SEMA3F*	Loss	High metastasis and poor survival	[14]	No	Yes	Yes
3p22.2	MLH1	Loss	Early stages of disease	[15]	Yes	Yes	Yes
3p14.2	*FHIT*	Loss	Early Event	[16]	No	Yes	No
3q26.32	PIK3CA	Gain	Poor prognosis	[17, 18]	No	No	Yes
3q26.33	SOX2	Gain	Metastasis,worse outcome, resistance to cisplatin	[19]	No	Yes	No
4q32.3	*PALLD*	LOH	Poor survival	[20]	No	Yes	No
4q32.3	*DDX60L*	LOH	Poor survival	[20]	No	Yes	No
4q35.2	*ING2*	LOH	Advanced stage	[21]	Yes	Yes	No
4q35.2	*FAT1*	LOH	Advanced tumour stage	[22]	No	Yes	No
7p11.2	*EGFR*	Gain	Poor prognostic	[23]	No	Yes	Yes
9p21.3	*CDKN2A*	Loss	High frequency of recurrences; early event in HNSCC progression	[24]	No	Yes	Gain
9p21.3	*CDKN2B*	Loss	Early event in HNSCC progression	[25]	No	Yes	Gain
11q13.3	*FADD*	Gain	Worse disease-specific survival	[26]	No	Yes	Yes
11q13.3	*ANO1*	Gain	Poor overall survival, metastases	[27, 28]	No	Yes	Yes
11q13.3	*CTTN*	Gain	Lymph node metastasis	[29]	No	Yes	Yes
11q13.3	*CCND1*	Gain	Presence of occult lymph node metastases, advanced clinical stage and shorter survival	[30, 31]	No	Yes	Yes
11q21	*MRE11A*	Loss	Reduced sensitivity to ionizing radiation in HNSCC	[32]	No	No	Yes
11q22.3	ATM	Loss	Reduced sensitivity to ionizing radiation in HNSCC	[32]	Yes	Yes	Yes
11q23.3	H2AFX	Loss	Reduced sensitivity to ionizing radiation in HNSCC	[32]	Yes	Yes	Yes
13q13.1	BRCA2	LOH	Poor patient outcome	[33]	Yes	Yes	Yes
13q14.2	RB1	LOH	Poor patient outcome	[33]	Yes	Yes	Yes
17p13.1	TP53	Loss	Nonresponse to neoadjuvant chemotherapy	[34]	Yes	Yes	Yes
18q21.2	*SMAD4*	Loss	Advanced stage and poor prognosis	[35]	No	Yes	No
18q23	*GALR1*	Methylation	Advanced stage and poor prognosis	[36, 37]	No	Loss	No
22q13.2	*CYB5R3*	Loss	Worse prognosis, decreased survival	[38]	Yes	No	No

**Fig. 4:**
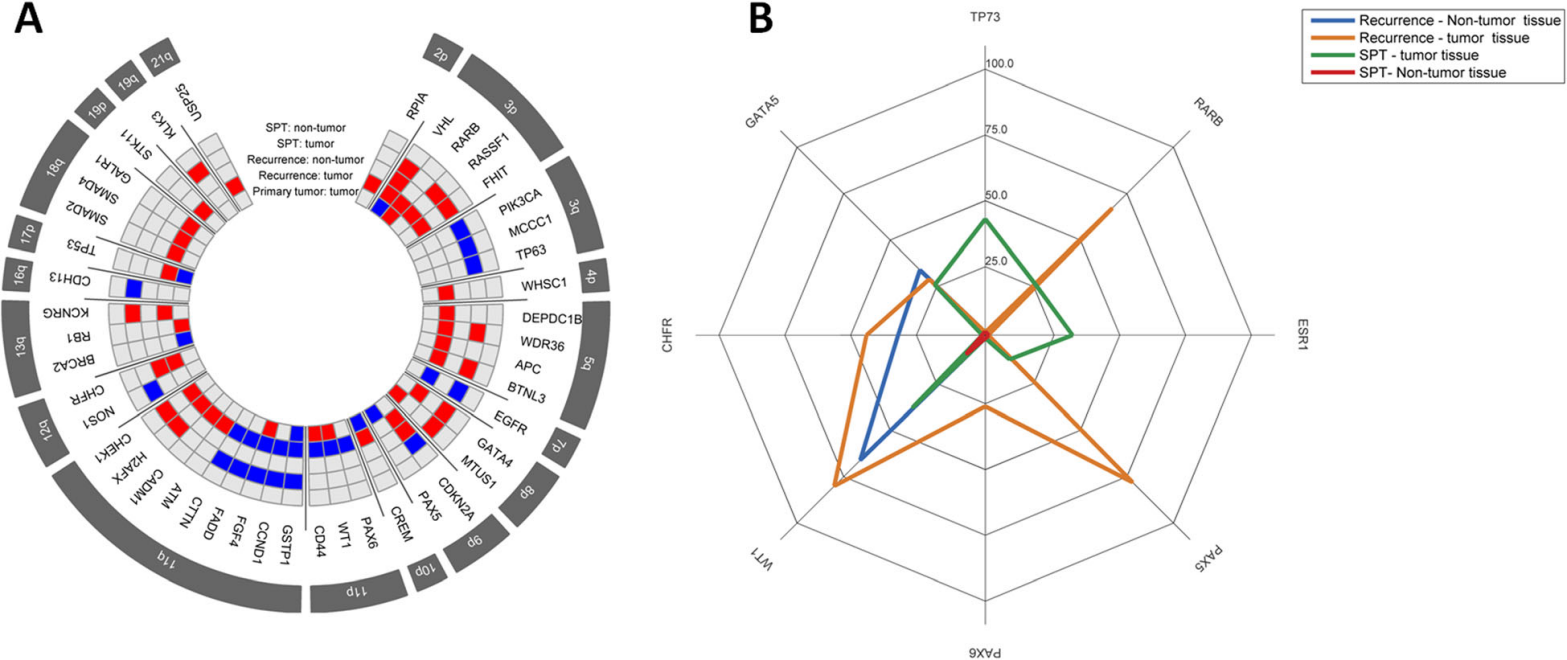
Results from MLPA and MS-MLPA. **a** Radar chart with copy number alterations detected by P248 and ME002 probemixes. Blue represents copy number gains and red copy number losses. **b** Radar chart with methylation status detected by ME002 probemix, highlighted the eight genes methylated in the samples of this patient. The scale represents the percentage of methylation detected

The MLPA and MS-MLPA results demonstrated that for all the analyzed genes the three tumors shared gain at 11q in the *GSTP1* gene (Fig. 4a). Considering recurrence and SPT we observed several shared imbalances, namely at 3p, 5q, 7p, 8p, 11q and 13q. We observed some different results between aCGH and MLPA/MS-MLPA due to the different sensibility of these techniques to detect low-level imbalances. The non clonal chromosome aberrations evidenced the genomic heterogeneity and complexity that is the reflex of chromosomal instability in the cellular population; however its frequency is relatively low, being for that often reported in the literature only the clonal chromosome aberrations [39].

Non-tumor sample of the recurrence presented genomic imbalances, namely losses at *VHL, CDKN2A* and *CHFR* genes (Fig. 4a). Primary tumor did not present methylation in any of the evaluated genes. Both recurrence and SPT presented *RARB, PAX5, WT1* and *GATA5* methylated (Fig. 4b). The highest number of gene promoter methylation was observed in the recurrent tumor sample. Tumor recurrence and the corresponding non-tumor sample exhibited *WT1, CHFR* and *GATA5* methylated (Fig. 4b). The presence of several genetic and epigenetic imbalances in both tumor and macroscopically non-tumor samples is indicative of the dissemination of cells with malignant features even without visible morphologic changes, remaining these cells after the resection of the tumor, and consequently increasing the risk of relapse. Thus, loss at *VHL* gene and *WT1* and *GATA5* gene promoter methylation seem to be important in the observed relapses*,* since these genetic and epigenetic imbalances were observed in both tumor and non-tumor tissue of recurrence and also in SPT tissue. These specific alterations have a role in the prognosis, relapse prediction and in the therapeutic response; however, a validation in a cohort of patients is needed. Clinical examination and histological assessment of surgical margin status alone have been considered not enough to predict the risk of recurrence [40], which was corroborated by our data of this patient. Moreover, a significant correlation between epigenetic profiling of clinically and histologically negative surgical margins and the development of SPT was also reported [41].

Our results are in agreement with the field cancerization theory, described in 1953 by Slaughter et al. [42] and with the cancer stem cell network model [43, 44], since the synergetic effect of alcohol and tobacco abuse lead to cumulative DNA alterations with higher progression to malignancy in the left side of the tongue (primary tumor), consequently, the genomic imbalances related to therapeutic resistance guarantees the persistence of cells with malignant features even after treatment which culminated in a local recurrence and also in the development of a SPT in a distant anatomic site (pharyngoesophageal region). The patient here described is a good example that genetic and epigenetic signatures should be taking into account in order to help in clinical management of OSCC patients.

## Conclusions

The clinical management of OSCC patients is complex and challenging. In the reported patient, we verified shared genomic imbalances, namely losses at 1p, 2p, 3p, 4q, 5q, 6q, 7q, 8p, 10p, 11q, 12p, 12q, 13q, 15q, 16p, 16q, 17p, 17q, 18q, 19p, 19q, 21q and Xp and gains at 3q, 7q, 14q and 15q, which are indicative of a common clonal origin for the relapses diagnosed. In the recurrent and SPT cells some imbalances seem to be acquired after treatment, such as in short arm of chromosomes 6, 7, 8 and 18. Losses at *VHL* gene and promoter methylation of *WT1* and *GATA5* genes seem to be important predictors of relapses. Further studies are needed in order to validate the putative biomarkers of diagnostic and prognostic highlighted with this patient.
